# Dispositional awe negatively predicts corruption via the sense of connectedness

**DOI:** 10.1002/pchj.737

**Published:** 2024-02-07

**Authors:** Liming Jiao, Li Luo

**Affiliations:** ^1^ Department of Economics and Management Neijiang Normal University Neijiang China; ^2^ Department of Education Science Neijiang Normal University Neijiang China

**Keywords:** corruption, dispositional awe, mediation effect, sense of connectedness

## Abstract

Corruption is a political and economic issue that has a detrimental impact on social and economic development. This study investigated the predictive effect of dispositional awe on corruption, and the underlying mechanisms from the perspective of connectedness. A sample of 548 (*N*_female_ = 371) individuals, aged between 16 and 71 years old (*M* = 21.18, *SD* = 3.39), was collected. Participants completed scales to measure dispositional awe, the sense of connectedness, corruption, and social desirability. Structural equation modelling and a bootstrap procedure were used to analyze the relationship between the variables. Results showed that dispositional awe negatively predicted corruption, including the tendencies for giving and accepting bribes, and this could be explained by the sense of connectedness, after controlling for the effect of social desirability. The findings highlight the significance of dispositional awe in relation to corruption, enhance our understanding of the underlying mechanisms connecting the two variables, and provide practical implications for the prevention of corruption.

## INTRODUCTION

Corruption is commonly defined as the misuse of entrusted power for personal gain (Hu et al., 2021; Kaufmann, 2004), which includes practices such as accepting and giving bribes, extortion, and embezzlement (Mishra, 2001). Corruption poses a significant threat to social and economic development (Chakravorty, 2019; Chang & Chu, 2006; Dincer & Gunalp, 2008; Hu et al., 2021), undermines institutional trust, and diminishes citizen well‐being (Kaufmann, 2004; Tay et al., 2014). The fight against corruption has emerged as a challenging and complex issue in the field of global management (Xu & Zhu, 2019). Corruption is characterized by prioritizing individual interests over legal, regulatory, and moral standards (Weisel & Shalvi, 2015). Therefore, reducing the emphasis on self‐interest is a crucial approach to combat corruption. Awe is a classic self‐transcendence emotion (Keltner & Haidt, 2003; Keltner & Piff, 2020; Stamkou et al., 2023; Stellar et al., 2017), which can encourage individuals to shift their attention from their own interests to the needs and welfare of the public (Jacobs & McConnell, 2022; Luo, Zuo, et al., 2022; Stellar et al., 2017). Can awe reduce corruption, and what is the mechanism underlying this association? In this study, we aim to answer these two questions, which will contribute to our understanding of the topic and provide new perspectives and empirical evidence on reducing corruption.

### The effect of awe on corruption

Awe occurs when individuals come across powerful and immense stimuli that go beyond their previous understanding, and that they need to accommodate to the present situation (Keltner & Haidt, 2003; Luo, Zuo, et al., 2022). Dispositional awe reflects individuals' enduring inclination to experience awe in daily life and their overall pattern of responses to it (Shiota et al., 2006). Previous studies have found that awe can compel individuals to adhere to social norms by encouraging them to integrate into collaborative social groups and engage in collective actions, and by inhibiting immoral behaviors (Bai et al., 2017; Keltner & Haidt, 2003; Luo, Zou, et al., 2022). A recent study has also shown that individuals who frequently experience moments of awe in their daily lives are more inclined to make sacrifices for the betterment of their group (Naclerio & Van Cappellen, 2021). All of these findings suggest that awe plays a critical role in shaping individuals' behavior.

Corruption is immoral that undermines collective interests and prioritizes individual gain (Weisel & Shalvi, 2015). Can dispositional awe negatively predict corruption? There have been limited empirical studies exploring this question. The affect infusion model describes emotion as having the function of infusion to influence cognitive processing and decision making (Forgas, 1995). Moreover, the moral foundations theory posits that moral emotions, including self‐transcendent emotions, play a crucial role in the development of the moral foundations that underlie political beliefs (Jacobs & McConnell, 2022; Valdesolo & Graham, 2014). Indirect evidence has found that awe can significantly influence individuals' values and shape their behaviors (Jiang et al., 2018; Paulson et al., 2021; Piff et al., 2015). On the one hand, awe can promote adherence to social conformity (Prade & Saroglou, 2023) and encourage more moral behavior (Bai et al., 2017; Luo, Zou et al., 2022). It is found that awe has the potential to facilitate harsher moral judgments and elevate moral consciousness (Tian, 2016), as well as to reduce immoral behaviors, such as aggression (Yang et al., 2016) and dishonesty (Luo, 2021). Awe also inspires individuals to donate more money to support public welfare (Luo, Zou, et al., 2022; Stamkou et al., 2023). On the other hand, awe can shape individuals' values, for example by enhancing the pursuit for spirit (Keltner & Haidt, 2003) and weakening the desire for money and material objects (L. Jiang et al., 2018). These findings support our hypothesis that dispositional awe may have a potential negative correlation with corruption.

### The mediation effect of connectedness

If dispositional awe can negatively predict corruption, what is the mechanism that underlies this association? Feelings as information theory posits that feelings can be a source of information, which affects subsequent judgments and decision making (Schwarz, 2012). Interpretative phenomenological analysis has indicated that the sense of connectedness (e.g. social connectedness and natural connectedness) is the main theme produced by awe, which reflects individuals' feelings of closeness to objects (Bonner & Friedman, 2011). Astronauts who viewed the Earth from space reported experiencing the emotion of awe, resulting in their identification with humankind and the planet as a whole (Yaden et al., 2016). Awe reduces interpersonal psychological distance and motivates individuals to connect themselves with the outside world, whether it be nature, humanity, or some other grand entity (Stellar et al., 2017). Therefore, awe can promote a sense of connectedness with the outside world, leading to a greater inclusion of beings in the self‐concept (Jiao & Luo, 2022; Luo, Zou, et al., 2022; Piff et al., 2015).

Though there have been no empirical studies about the relationship between connectedness and corruption, relevant theories and studies provide the basis for this association. Social control theory proposes that being connected to the outside world can help individuals control themselves from engaging in immoral or deviant behaviors (Costello & Laub, 2020). Empirical research has also demonstrated that the sense of connectedness could increase prosocial behavior (Feng et al., 2020; Luo, Yang, et al., 2022; Rosa et al., 2018; Yang et al., 2016) and control immoral or criminal behavior (Hirschi, 2008; Jiang et al., 2020; Özbay & Özcan, 2006). Stuart and Taylor (2021) investigated the effect of social connectedness on crime in America from 1970 to 2009 and found that it could reduce incidents of rape, murder, robbery, assault, burglary, and motor vehicle theft. A connection to nature also increases prosocial and pro‐environmental behavior (Barbaro & Pickett, 2016; Castelo et al., 2021; Jacobs & McConnell, 2022; Rosa et al., 2018) and reduces aggressive behavior (Poon et al., 2016). Therefore, corruption may be controlled by connectedness. Meanwhile, we hypothesized that connectedness may mediate the relationship between dispositional awe and corruption.

### Study overview

Corruption is a serious and widespread social phenomenon (Tay et al., 2014). While many studies have attempted to address it (Bautista‐Beauchesne, 2022; Bertot et al., 2010), few have investigated the role of self‐transcendent emotions, such as awe, in reducing it and the underlying mechanism behind this association. Therefore, the aim of this study is to investigate the impact of dispositional awe on corruption, along with the mechanisms that connect these two variables, using a cross‐sectional approach. We measured the tendencies to give and accept bribes as the dependent variables because these are typical indicators of corruption (Mishra, 2001; Salmon & Serra, 2017). The exploration of this issue helps deepen our understanding of the relationship between awe and corruption and provides new theoretical and empirical foundation for combating corruption.

## METHODS

### Participants

This is a cross‐sectional study. We used random sampling to recruit participants from four universities in China and asked them to invite their families or classmates to take part through the snowball sampling method.

We collected data from October 2022 to February 2023 via online self‐report questionnaires. This study was conducted in accordance with the Declaration of Helsinki, and approved by the internal ethics committee of Neijiang Normal University (protocol number: 20220001). We obtained informed written consent from participants prior to them starting to answer the questionnaire online. Participants were also informed that the data would be confidential and that only the researchers in this study could access information about them. Participants were compensated with ¥5 (approximately $0.73) after completing the questionnaires. To ensure the validity of data, we informed the participants ahead of time that the fee would only be paid once the researchers approved the data quality.

The Monte Carlo Power Analysis for Indirect Effects Application was used to determine the sample size (https://schoemanna.shinyapps.io/mc_power_med/) (Schoemann et al., 2017). At least 109 participants were needed to reach a power of .80. The online survey included 548 individuals (*N*_females_ = 376) who participated this study, and their age ranged from 16 to 72 years, with an average age of 21.18 ± 3.39 years. Their careers included students, doctors, programmers, teachers, office workers, and so on.

### Materials

#### Dispositional awe

We used the awe scale in the Dispositional Positive Emotion Scale (DPES‐awe) to measure participants' dispositional awe (Shiota et al., 2006). DPES‐awe is a unidimensional scale that includes six items, such as “I often feel awe,” “I seek out experiences that challenge my understanding of the world.” Participants were required to rate their level of agreement for each item on a 7‐point Likert scale, ranging from 1 (*strongly disagree*) to 7 (*strongly agree*). A higher score represents an increased frequency of experiencing awe in daily life. Cronbach's alpha coefficient of DPES‐awe in this study was .85.

#### Sense of connectedness

The sense of connectedness was assessed by five items (Luo, Yang, et al., 2022), such as “I think I belong to a larger entity,” “Humankind is a whole.” This is a unidimensional scale, and participants reported their agreement for each item on Likert‐type scales ranging from 1 (*strongly disagree*) to 7 (*strongly agree*). A higher score indicates that participants experienced a greater sense of connectedness in their daily lives. Cronbach's alpha coefficient in this study was .84.

#### Corruption

There were two indicators of corruption observed in this study. First, participants' propensity to give bribes in business was assessed using the Intercultural Business Corruptibility Scale (Leong & Lin, 2009). This unidimensional scale consists of 14 items, and participants respond to each item on a 5‐point scale, with 1 indicating “*strongly disagree*” and 5 indicating “*strongly agree*.” A higher score indicates greater propensity to give bribes. Because this scale was being used in China for the first time, we conducted confirmatory factor analysis (CFA) and found that the factor loads of seven items were below .40. Therefore, we retained the remaining seven items for further analysis. The model fitting was plausible, χ^2^ (14) = 40.70, comparative fitting index (CFI) = .98, normed fit index (NFI) = .96, goodness‐of‐fit index (GFI) = .98, root mean square error of approximation (RMSEA) = .06, and standardized root mean square residual (SRMR) = .03. All the factor loads were larger than .49. In this study, the Cronbach alpha coefficient was .81.

The second indicator of corruption was the willingness to accept a bribe. Participants were presented with a hypothetical scenario in which a driver violated traffic laws and wanted to bribe a traffic police officer with money under the table to reduce punishment (Zhao et al., 2021). Participants were asked to imagine themselves as the police officer and respond to two questions on a 7‐point Likert scale ranging from 1 (*not at all likely*) to 7 (*highly likely*): “How likely are you to help this driver?” and “How likely are you to accept private payment from this driver?” The two questions were summed and averaged to ascertain the participants' inclination to accept a bribe.

In addition, bribery may be influenced by social desirability (Zhao et al., 2021). Therefore, we assessed social desirability as the control variable, adopting the Marlowe–Crowne Social Desirability Scale (Crowne & Marlowe, 1960). There are 33 items, which are answered binarily (0 – no, 1 – yes). Cronbach's alpha of this scale was .77 in the present study.

### Data analysis

Descriptive statistics and Pearson correlation coefficients were conducted in SPSS 23.0, and the structural equation model was estimated in AMOS 23.0 (SPSS Inc, Chicago, IL, USA). All data have been made publicly available via The Open Science Framework repository, named awe and corruption, and can be accessed at https://osf.io/rnx4s/.

## RESULTS

### Common method variance

Because all the data in the current study were self‐reported and collected during the same period, we need to consider the possibility of common method bias (CMB). To address this concern, we applied Harman's one‐factor method, a well‐established approach to detecting CMB (Podsakoff et al., 2012). The results of principal component analysis showed that all the variables produced 15 distinct factors with characteristic roots greater than one, which accounted for 55.62% of the total variance. The first factor only accounted for 14.59% of the variance, which is lower than the criterion of 40% (Podsakoff et al., 2012). Therefore, CMB is not a major issue in the present study.

### Descriptive statistics and correlations

The descriptive statistics and the Pearson's correlations of the variables are presented in Table 1.

**TABLE 1:** Descriptive statistics and Pearson's correlations for the variables (*N* = 548).

Variables	*M* ± *SD*	1	2	3	4	5
1. Dispositional awe	5.03 ± .93	1				
2. Connectedness	5.78 ± .88	.49***	1			
3. Business corruption tendency	2.75 ± .36	−.24***	−.47***	1		
4. Willingness to accept bribes	2.77 ± 1.59	−.17***	−.31***	.43***	1	
5. Social desirability	18.52 ± 5.26	.38***	.25***	−.21***	−.28***	1

*** *p* < .001.

The results demonstrate that dispositional awe has a positive correlation with the sense of connectedness (*r* = .49, *p* < .001). Furthermore, both variables are negatively correlated with corruption tendency (*r* = −.32, *p* < .001; *r* = −.17, *p* < .001, for dispositional awe and connectedness, respectively) and the willingness to accept bribes (*r* = −.37, *p* < .001; *r* = −.31, *p* < .001, for dispositional awe and connectedness, respectively).

### Mediation effects analysis

**FIGURE 1:**
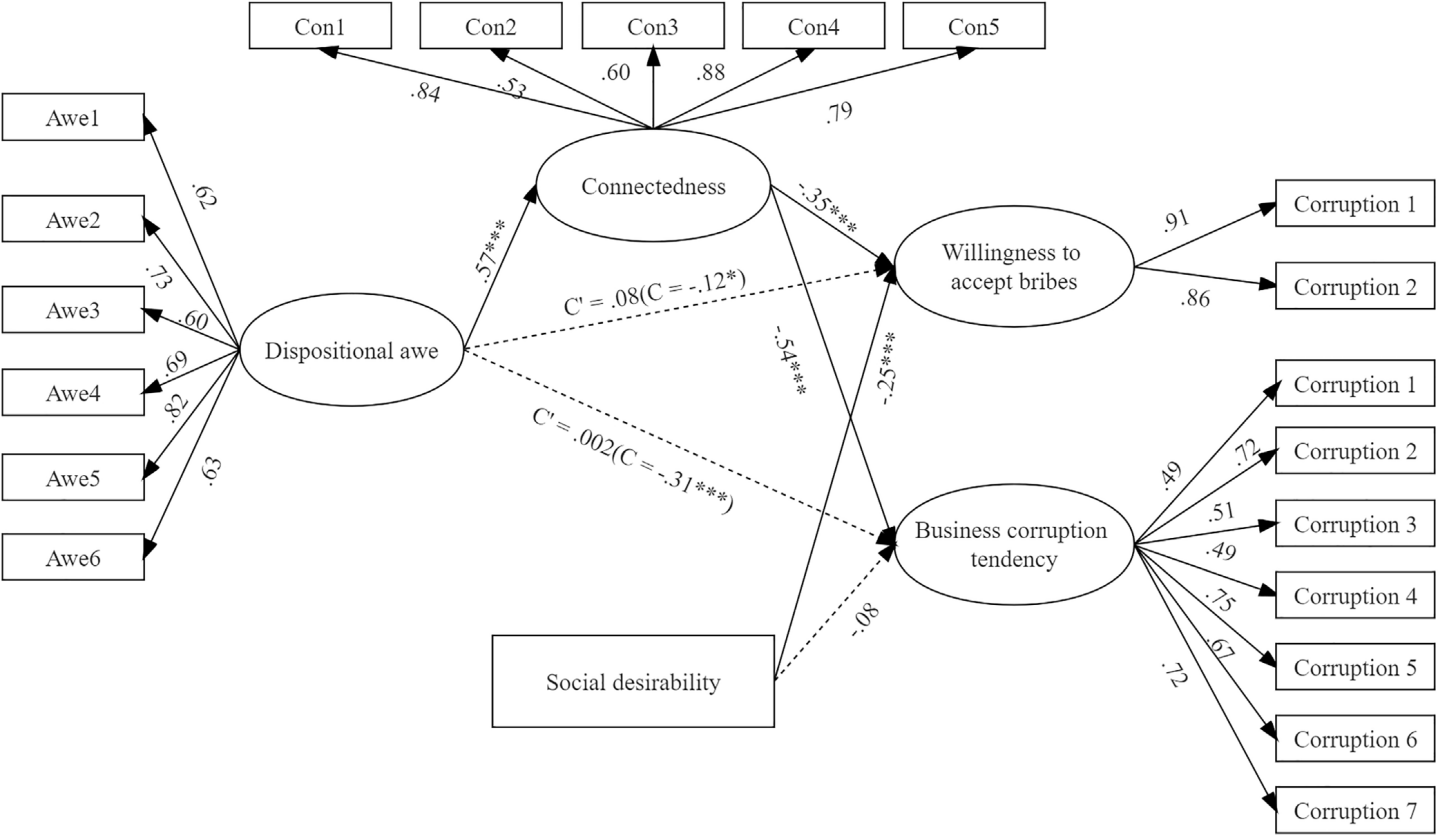
Mediation model for the effect of dispositional awe on corruption tendency (*N* = 548). All path coefficients were standardized. The dotted line indicates the insignificant path coefficient. The model fitting was satisfactory, χ^2^ (180) = 524.46, comparative fitting index = .93, normed fit index = .90, goodness‐of‐fit index = .91, root mean square error of approximation = .06.

We performed two mediation analyses with AMOS 23.0 to determine whether connectedness mediated the relationship between dispositional awe and corruption tendency and willingness to accept bribes, respectively. The structural equation model, along with a parametric bootstrap procedure consisting of 5000 replications, was used to calculate the 95% bias‐corrected confidence intervals (CIs) for the indirect effects of the parameters and standard errors. The model, which is displayed in Figure 1, featured dispositional awe as the input variable, the sense of connectedness as the mediator, corruption tendency and willingness to accept bribes as the outcome variables, and social desirability as the control variable.

The results indicate that the total effect of dispositional awe on business corruption tendency was significant (β = −.31, *p* < .001, 95% CI [−.42, −.19]), while the direct effect was not (β = .002, *p* = .941, 95% CI [−.12, .13]). Furthermore, the sense of connectedness was associated with a decrease in business corruption tendency (β = −.54, *p* < .001, 95% CI [−.65, −.43]). Notably, there was a significant indirect effect of awe on business corruption tendency through the mediation of connectedness (mediation effect = −.31, 95% CI [−.40, −.24], *p* < .001), even after controlling for the effect of social desirability. These findings imply that the reduction in corruption caused by awe is attributable to the strengthening of the sense of connectedness.

In addition, the results show that the total effect of dispositional awe on the willingness to accept bribes was significant (β = −.12, *p* = .03, 95% CI [−.23, −.01]), while the direct effect was not (β = −.08, *p* = .225, 95% CI [−.05, .21]). Furthermore, the sense of connectedness was observed to be negatively associated with the willingness to accept bribes (β = −.35, *p* < .001, 95% CI [−.47, −.24]). After controlling for the effect of social desirability, a significant indirect effect of awe on the willingness to accept bribes through connectedness was observed (mediation effect = −.20, 95% CI [−.29, −.14], *p* < .001). Thus, the results suggest that dispositional awe weakens the willingness to accept bribes by increasing the sense of connectedness.

## DISCUSSION

The effective control of corruption can lead to beneficial outcomes for social and economic development. In this study, we employed a cross‐sectional design to examine the association between dispositional awe and corruption. We also investigated the mechanisms behind this relationship from the perspective of connectedness. We found that dispositional awe negatively predicts corruption and that this relationship is mediated by the sense of connectedness.

### The effect of dispositional awe on corruption

Corruption, namely violating social norms and laws, is both immoral and illegal, as individuals abuse public rights for their own interests (Kaufmann, 2004). Based on previous research, our current study found that dispositional awe is negatively associated with corruption. In other words, individuals who frequently experience the feeling of awe in their daily lives are less likely to engage in giving or accepting bribes. These findings are consistent with previous studies that have demonstrated that dispositional awe has a positive impact on moral behavior and the ability to decrease unethical conduct (Jiao & Luo, 2022; Piff et al., 2015; Stamkou et al., 2023; Yang et al., 2016). Furthermore, this work demonstrates that the affect infusion model (Forgas, 1995) and moral foundations theory (Jacobs & McConnell, 2022) are useful frameworks for understanding the predictive effect of dispositional awe on corruption. Awe is a profound emotional experience that has the power to assist individuals in overcoming self‐centeredness and psychological entitlement, enabling them to transcend their preoccupation with self‐interest (Luo, Zou, et al., 2022; Piff et al., 2015; van Elk et al., 2019; Zhang, 2022). As a moral emotion associated with self‐transcendence, awe can influence people's values, leading to a greater focus on spiritual pursuits (Joye & Dewitte, 2016) and a reduced desire for material wealth and money (Jiang et al., 2018). All these factors contribute to a stronger concern for societal well‐being (Bai et al., 2017) and adherence to social norms (Prade & Saroglou, 2023), consequently decreasing unethical and illegal behaviors. Our research enhances current knowledge of the relationship between awe and social behavior by exploring the inhibitory effect of dispositional awe on misconduct. Moreover, it is suggested that fostering awe can effectively contribute to reducing individual‐level corruption.

### The mediation effect of connectedness

In line with feelings as information theory (Schwarz, 2012) and social control theory (Hirschi, 2008), we have discovered that the sense of connectedness can explain the association between dispositional awe and corruption. To our knowledge, this is the first time that the mechanism underlying this relationship has been identified from the perspective of connectedness. Awe is experienced when individuals are confronted with vast objects (Keltner & Haidt, 2003), which causes them to perceive themselves as part of a larger entity (Bai et al., 2017; Piff et al., 2015). Consequently, connectedness with the outside world, including other people or nature, is an important feeling when awe is generated, and it can control self‐centered impulses and shape individuals' behavior (Austin, 2010). Establishing a sense of connection with other people, such as parents, can help establish social control and deter deviant behavior. This is because individuals are generally reluctant to jeopardize their relationships. In contrast, a lack of adequate social connectedness may lead individuals to act without moral constraints on their behavior. In addition, connecting with nature also inspires individuals to broaden their horizons and transcend their mundane concerns (Castelo et al., 2021). Another study found a positive correlation between awe and an increased sense of connectedness, which subsequently reduced narcissism (van Mulukom et al., 2020). Narcissism is characterized by a desire for entitlement, grandiose fantasies, and the need for admiration (Montoro et al., 2022), which can decrease concern for the public's welfare and needs (McGregor et al., 2013). All the evidence supports our current findings that dispositional awe is positively correlated with connectedness, thereby predicting a decreased inclination toward corruption.

### Implications

This work extends previous studies about awe and social behavior, contributing to our knowledge on awe and providing theoretical and practical implications for reducing corruption. Corruption is a destructive social issue and a major global challenge. Drawing on the affect infusion model and moral foundations theory, we examined how dispositional awe predicts corruption. Additionally, we explored the mechanism through which dispositional awe influences corruption, considering the perspectives of feelings as information theory and social control theory. Previous research has demonstrated that dispositional awe can promote prosocial behaviors (Jiao & Luo, 2022; Piff et al., 2015), such as helping and sharing, while also inhibiting antisocial behaviors, such as aggression (Yang et al., 2016) and dishonesty (Luo, 2021). However, our study reveals that awe can also negatively predict engagement in illegal activities. Individuals with higher dispositional awe are less likely to violate laws and regulations, which contributes to maintaining social order and harmony. Further analysis suggests that experiencing dispositional awe can lead to a stronger sense of connectedness and ultimately decrease instances of corruption. These findings deepen our understanding of awe, as well as its relationship with social behavior. At the same time, the present study also offers us a novel practical approach to combating corruption from the perspective of self‐transcendent emotion. As the ancient Chinese saying goes, “With awe in mind, one knows where to stop.” Existing studies have identified magnificent natural landscapes, solemn memorial sites, and observing noble behaviors in others as effective triggers for inducing awe (Bai et al., 2017; Gordon et al., 2017; Keltner & Haidt, 2003).

### Limitations and directions for future research

There are several limitations to our research that require further study in the future. First, this empirical study is cross‐sectional, we need to be cautious in inferring the causal relationship amoung awe, connectedness, and corruption. Dispositional awe reflects the tendency to experience the feeling of awe in daily life, which is not manipulated in this study. Therefore, additional research is needed to replicate these findings using experimental approaches. Prior studies have found that methods such as watching videos, recalling‐and‐writing tasks, and going on field trips are all effective in inducing awe (Luo et al., 2021; Piff et al., 2015).

Second, we employed multiple indexes of corruption and, after accounting for the social desirability effect, discovered that connectedness could explain the association between dispositional awe and corruption. Nevertheless, the elimination of the social desirability effect proved to be challenging. Thus, future research should incorporate objective indicators of corruption to mitigate the impact of social desirability.

Third, although the main forms of corruption are accepting and giving bribes, it also takes other forms, such as extortion and embezzlement (Mishra, 2001). It is necessary to explore the influence of awe on other facets of corruption in the future, as this will help illuminate the breadth and boundaries of awe's effects on corruption. Additionally, we primarily measured the intention of corruption rather than actual corrupt behavior. Although intention to implement an action may be a valid predictor of that behavior, there are several factors that can hinder the translation of behavioral intentions into actual behavior (Gosling & Williams, 2010). Therefore, future research should measure actual corrupt behavior in order to better understand the impact of awe on corrupt behavior. In a bribery game, three roles are assigned – public official, private citizen, and member of society. The game begins with the private citizen having to decide whether to offer a bribe to the public official. Then, it is up to the official to determine whether to accept the bribe. If the bribe is indeed offered and accepted, both the citizen and the official gain money, but this comes at the expense of the third member of society (Salmon & Serra, 2017).

## CONCLUSIONS

This work illustrates the impact of dispositional awe on corruption and its underlying mechanisms. The results shows that dispositional awe plays a role in corruption, and the sense of connectedness mediates this relationship. This study has practical implications for the promotion of integrity and self‐discipline among individuals. It underscores the importance of cultivating the experience of awe as a means to combat corruption.

## FUNDING INFORMATION

This research was funded by the Humanity and Social Science Youth Foundation of the Ministry of Education (17XJC190004; Li Luo).

## CONFLICT OF INTEREST STATEMENT

The authors declare no conflict of interest. The funder had no role in the design of the study; in the collection, analyses, or interpretation of data; in the writing of the manuscript; or in the decision to publish the results. The authors declare that the research was conducted in the absence of any commercial or financial relationships that could be construed as a potential conflict of interest.

## INSTITUTIONAL REVIEW BOARD STATEMENT

The study was conducted in accordance with the Declaration of Helsinki, and approved by the internal ethics committee of Neijiang Normal University (protocol number: 20230001).

## ETHICS STATEMENT

This study obtained ethics approval from the internal ethics committee of Neijiang Normal University (Protocol number: 20220001). We obtained informed written consent from participants prior to them starting to answer the questionnaire online.

## Data Availability

All data and analysis code have been made publicly available via The Open Science Framework repository, named data for awe and corruption, and can be accessed at https://osf.io/rnx4s/.
